# A newly identified berberine derivative induces cancer cell senescence by stabilizing endogenous G-quadruplexes and sparking a DNA damage response at the telomere region

**DOI:** 10.18632/oncotarget.5521

**Published:** 2015-10-08

**Authors:** Yun-Xia Xiong, Hua-Fei Su, Peng Lv, Yan Ma, Shi-Ke Wang, Hui Miao, Hui-Yun Liu, Jia-Heng Tan, Tian-Miao Ou, Lian-Quan Gu, Zhi-Shu Huang

**Affiliations:** ^1^ School of Pharmaceutical Sciences, Sun Yat-sen University, Guangzhou 510006, P.R.China; ^2^ Department of Medical Science, Shunde Polytechnic, Foshan 528333, P.R.China

**Keywords:** berberine derivative, telomeric G-quadruplex, DNA damage, cancer cell senescence

## Abstract

The guanine-rich sequences are able to fold into G-quadruplexes in living cells, making these structures promising anti-cancer drug targets. In the current study, we identified a small molecule, Ber8, from a series of 9-substituted berberine derivatives and found that it could induce acute cell growth arrest and senescence in cancer cells, but not in normal fibroblasts. Further analysis revealed that the cell growth arrest was directly associated with apparent cell cycle arrest, cell senescence, and profound DNA damage at the telomere region. Significantly, our studies also provided evidence that Ber8 could stabilize endogenous telomeric G-quadruplexes structures in cells. Ber8 could then induce the delocalization of TRF1 and POT1 from the telomere accompanied by a rapid telomere uncapping. These results provide compelling insights into direct binding of telomeric G-quadruplexes and might contribute to the development of more selective, effective anticancer drugs.

## INTRODUCTION

Guanine-rich nucleic acids have the ability to fold into a four-stranded secondary structure known as a G-quadruplex, and novel anti-cancer therapy strategies binding and stabilizing these structures have attracted significant attention in recent years. The availability of a new G-quadruplex antibody, BG4, for quantitatively visualizing G-quadruplexes in both cancer cells and tissues has enhanced the attractiveness of this strategy [1, 2]. Some studies on G-quadruplex's ligands in living cells also highlight the potential for binding and stabilizing G-quadruplexes in cells [3, 4].

The human telomeric DNA contains repetitive TTAGGG sequences in the overhang; these repeats can form G-quadruplex structures [5]. This G-overhang acts as a substrate for the catalytic subunit of telomerase, which is a reverse transcriptase with an RNA component required for telomere extension [6]. The telomere terminus is protected from degradation or illegitimate recombination by a T-loop, which forms through strand invasion of the 3′-overhang into the duplex part of the telomere [7]. The T-loop is stabilized by a six-subunit protein complex called shelterin, containing TRF1, TRF2, POT1, TIN2, TPP1, and Rap1. Among them, TRF1, TRF2, and POT1, directly recognize TTAGGG repeats [8]. G-quadruplex small molecular ligands have the ability to interfere with telomere function in multiple ways: the ligands can inhibit telomerase activity by blocking the binding of telomerase with G-overhang [9]; the ligands can dissociate the shelterin protein complex from the telomere and lead to telomere uncapping and degradation [10–12]; and the ligands can interfere with telomere replication by impairing replication fork progression [13, 14]. Several types of G-quadruplex ligands can counteract the extension of telomere in anticancer therapy [15–17]. However, challenges still exist in developing G-quadruplex ligands because of the non-validated target location and insufficient drug-like properties.

Berberine, an isoquinoline alkaloid isolated from Chinese herbs, has shown anticancer potential against a wide range of human cancer cells and minimal cytotoxicity in normal cells [18–21]. Berberine and its derivatives could bind directly with the G-quadruplex by external stacking to G-tetrad [22–24] and have poor selectivity for duplex DNA [25]. 9-substituted berberines with longer side chains and terminal amino groups were previously synthesized by our group and were found to have significant telomeric G-quadruplex-binding ability and improved selectivity compared to berberine [26–29]. In the present study, we firstly applied several screening methods to evaluate the interaction of our in-house library of berberine derivatives (Supplementary Figure S1) with telomeric G-quadruplexes and to identify a promising ligand. We found that Ber8, a newly synthesized berberine derivative with a side chain of chlorohexyl group at the 9-position, had strong interaction with telomeric G-quadruplexes and could effectively induce acute cell growth arrest in cancer cells. Moreover, the inhibition of cancer cell growth by Ber8 was associated with apparent cell cycle arrest, cell senescence, and profound DNA damage at telomere regions. Further mechanic studies revealed that Ber8 could not only increase endogenous telomeric G-quadruplexes in cancer cells but also delocalize TRF1 and POT1 from the telomere and induce telomere uncapping.

## RESULTS

### Screening of effective berberine derivatives as G-quadruplex ligands for cancer cell treatment

To evaluate the interaction between berberine derivatives and G-quadruplexes, FRET assays were firstly applied. FRET can evaluate the thermo-stability of telomeric G-quadruplex DNA (HTG21). The structures of the berberine derivatives were listed in Supplementary Figure S1. The changes in melting temperatures (Δ*T*_m_) of the G-quadruplex DNA were calculated from the original FRET data and shown in a column graph (Supplementary Figure S2A). All the derivatives exhibited stabilizing effects on the HTG21 oligomer (the Δ*T*_m_ values ranged from 5°C ~ 22°C). This finding was agreed with our previous reports that introduction of a side chain (especially with a positively charged aza-aromatic terminal group) on the 9-position of the berberine can lead to significant increased stabilization of G-quadruplex DNA [26–28]. In addition, if 10°C of Δ*T*_m_ was set as a threshold to determine effective G-quadruplex stabilization, the derivatives ber7 to ber22 could be selected as G-quadruplex stabilizers.

In addition to the effect on the thermo-stability of DNA, the binding of compounds to DNA is also an important parameter for identifying an effective G-quadruplex ligand. We further evaluated the binding ability and selectivity of berberine derivatives with telomeric G-quadruplex DNA (HTG22) and hairpin DNA by using SPR. After fitting the original data from SPR, the binding constants (*K*_D_) could be calculated. As shown in Supplementary Figure S2B and Supplementary Table S1, most of the compounds demonstrated strong binding abilities with the HTG22, with the exception of BBR, ber1 to ber7, and ber9. The *K*_D_ values of the effective ligands were at a level of 10^−5^ to 10^−6^ mol/L. More importantly, the effective compounds possessed good selectivity for the HTG22 against the hairpin DNA. Due to their effective stabilization and selective binding ability, compounds Ber8 and ber10 - ber22 were chosen for further investigation.

**Figure 1:**
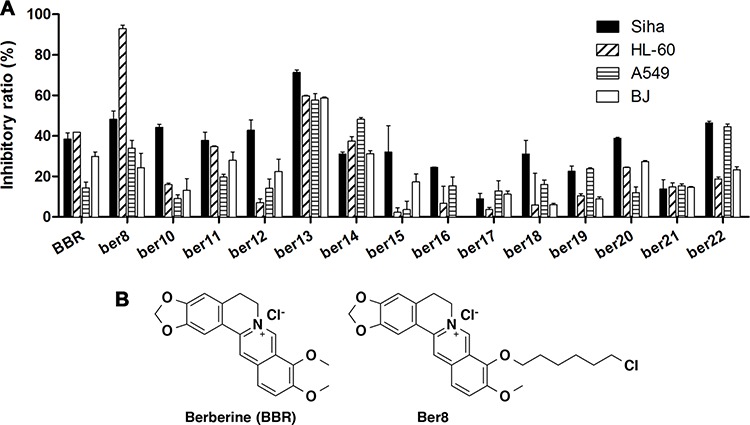
The inhibitory effects of compounds on tumor cell and normal cell proliferation **A.** Cells were exposed to 20 μM compounds for 48 h. After MTT detection, the inhibition rates were calculated using the following equation: inhibitory ratio = (1 – OD_drug_ / OD_control_)*100%. **B.** The structures of Ber8 and BBR.

To identify effective G-quadruplex ligands with anti-tumor activity, MTT assays that assess cytotoxicity were applied. Human cervical tumor cells Siha, human non-small cell lung cancer cells A549, human promyelocytic leukemia cells HL60, and normal human cells BJ were tested. We found that Ber8 exhibited the strongest inhibitory effects on the tumor cells and a weak inhibitory effect on normal cells (Figure 1A). Therefore, the compound Ber8 may be a good candidate for further cellular studies, and BBR was used as a compound control. The structure of Ber8 and BBR were shown in Figure 1B.

### Induction and stabilization of telomeric G-quadruplex DNA by Ber8 *in vitro*

**Figure 2:**
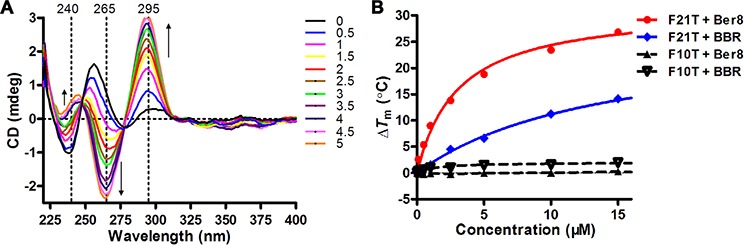
Induction and stabilization of telomeric G-quadruplex DNA by Ber8 *in vitro* **A.** CD spectra of HTG21 (5 μM) after addition of **Ber8** at increasing concentrations (from 0 to 5 mol equiv.) in 10 mM Tris-HCl buffer (pH 7.4). Changes in ellipticity at characteristic wavelengths (240 nm, 265 nm, and 295 nm) were indicated by black arrows. **B.** Concentration-dependent melting curves (Δ*T*_m_ *vs*. ligand concentration) for ligand Ber8 on F21T and F10T. The concentrations of F21T and F10T were both at 0.2 μM. The *T*_m_ values of annealed F21T and F10T were 60°C and 64°C, respectively. Δ*T*_m_ = *T*_m_ (DNA + ligand) − *T*_m_ (DNA).

The interaction between Ber8 and the telomeric G-quadruplex was further examined by using CD spectroscopy and FRET assays. In the absence of potassium, sodium, or any other cations, the CD spectrum of randomized HTG21 oligonucleotide had a negative peak at 238 nm, a major positive peak at 257 nm, a minor negative peak at 280 nm, and a positive peak at near 295 nm (Figure 2A, black line). Titration of Ber8 into HTG21 induced the formation of an antiparallel G-quadruplex in a dose-dependent manner, exhibiting minor positive peak at 240 nm, a negative peak at 265 nm, and a major positive peak at 295 nm (Figure 2A). In addition, as shown in Figure 2B, Ber8 could enhance the melting temperature (*T*_m_) of HTG21 in dose-dependent manner but did not increase the *T*_m_ value of the hairpin duplex DNA (F10T). Combining with the previous data, Ber8 could selectively bind and stabilize the telomeric G-quadruplex *vs*. duplex DNA. As a control, BBR could only induce a weak increase on *T*_m_ of either the HTG21 DNA or the F10T DNA (data not shown).

### Ber8 may inhibit tumor cell proliferation and induce DNA damage and repair

**Figure 3:**
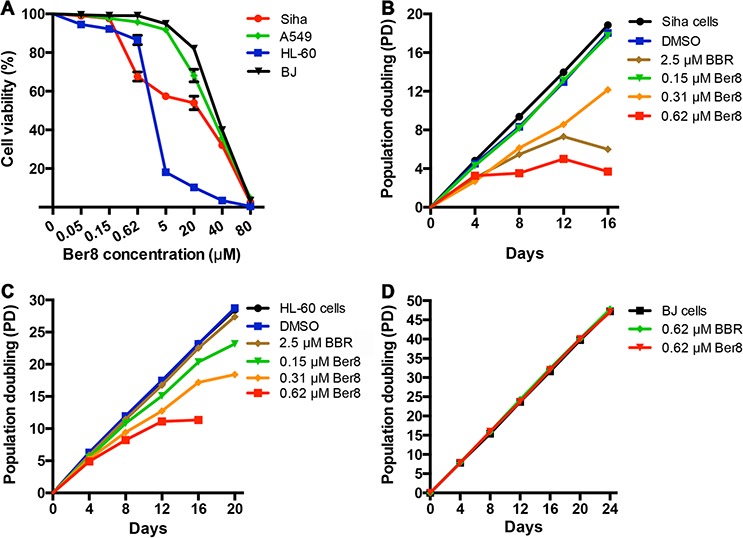
Cancer cell-growth suppression by Ber8 and BBR **A.** Cell growth inhibition curves of cancer cells (Siha cells, A549 cells, and HL-60 cells) and normal cells (BJ fibroblasts) after a 48-h treatment with Ber8. The data were reported as the percentage of growing cells with respect to that of untreated cells. The data represented the means of three independent experiments with the s.e.m. **B, C.** and **D.** Proliferation curves of Siha (B), HL-60 (C) cells and BJ fibroblasts (D) treated with Ber8, BBR, or 0.1% DMSO. The cells were counted and passaged at the indicated times, and population doubling (PD) values were calculated from the cell amounts.

The effects of Ber8 on cell proliferation in 48 h were assessed on Siha, A549, HL60, and BJ cells. Treatment of cells with increasing concentrations of Ber8 led to a remarkable arrest of cell growth in a dose-dependent manner in Siha and HL60 cells, but not in A549 and BJ cells (Figure 3A). The half-concentrations of inhibition (IC_50_) of Ber8 on these cells were 28.8 ± 1.0 μM (BJ), 6.0 ± 0.9 μM (Siha), 1.7 ± 0.5 μM (HL60), and 18.6 ± 1.3 μM (A549), respectively. The cellular uptake amounts of Ber8 were shown in Supplementary Table S2. Ber8 could be taken up by these cells, the uptake amounts varied from different cells. In brief, the uptake amounts was highest in HL-60 cells, the uptake amounts in Siha was higher than those in A549 cells. Thus, the different inhibitory effects of Ber8 on different cells might come from different uptake efficiency. In addition, the growth inhibitory effects of Ber8 were largely improved compared with BBR (Supplementary Table S3).

To evaluate the effects of Ber8 on the proliferation of cancer cells and normal somatic cells over a relatively long-term period, subcytotoxic concentrations (0.15, 0.3, and 0.6 μM) of Ber8 were used to treat Siha cells, HL-60 cells, and BJ fibroblasts, respectively. After 16 days of treatment, growth arrest could be observed in both Siha cells and HL-60 cells at 0.6 μM (Figure 3B and 3C). In contrast, BJ cells treated with Ber8 at the same concentration demonstrated little change in cell growth (Figure 3D). BBR exhibited a much weaker inhibitory effect on the proliferation of cancer cells (Figure 3B and 3C). These results showed that Ber8 could effectively and selectively inhibit the proliferation of cancer cells.

**Figure 4:**
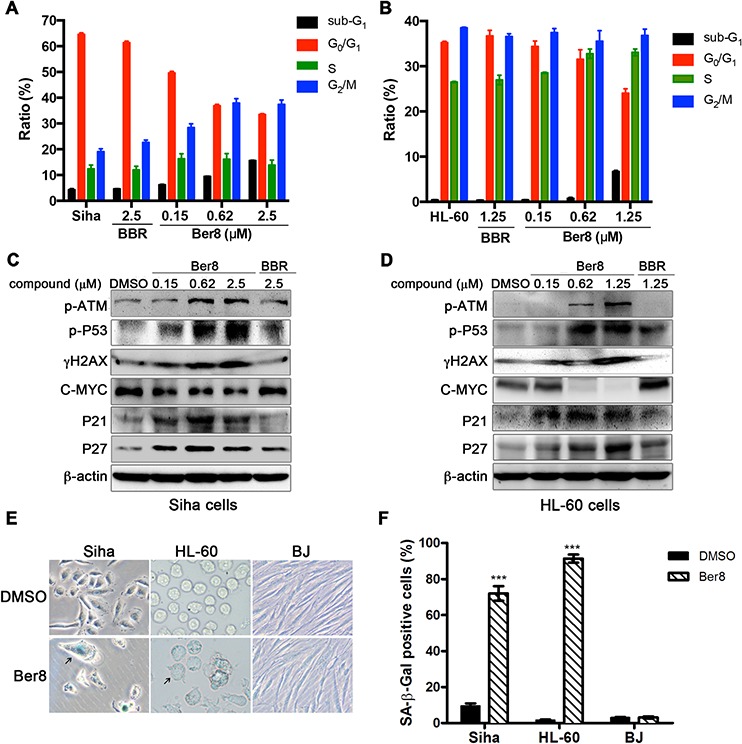
The effects of Ber8 on cellular events **A.** and **B.** Cell cycle analysis after propidium iodide (PI) staining after 48-h treatment with Ber8, BBR, or 0.1% DMSO in Siha cells (A) and HL-60 cells (B). The percentage of cells in different phases of the cell cycle was analyzed by EXPO32 ADC software. The data represented means of three independent experiments with the s.e.m. **C.** and **D.** Expression of pathway-related proteins in Siha cells (C) and HL-60 cells (D) treated with Ber8, BBR, or 0.1% DMSO for 48 h. **E.** Expression of SA-β-Gal in Siha, HL-60, and BJ cells after 16-day treatment with 0.62 μM of Ber8. The black arrow indicates a typical image of SA-β-Gal-positive cells. Original magnification, 40×. **F.** The percentage of SA-β-Gal-positive cells comparing with the total number of cells. The senescent cells were counted under an inverted microscope in three random fields. ***, *P* < 0.0001 compared with DMSO.

To further examine the effects of Ber8 on cellular events, we applied a panel of cellular assays on Siha and HL60 cells. Firstly, a flow cytometry assay determining the percentage of cells in each phase of the cell cycle was conducted. As shown in Figure 4A, after treatment with Ber8, Siha cells in the sub-G_1_ phase showed an observable increase from 4.4% to 15.6%, and cells in the G_2_/M phase increased from 19.1% to 37.5%. Simultaneously, an obvious decrease of cells in the G_0_/G_1_ phase from 64.6% to 33.5% was also observed. On the other hand, Ber8 tended to accumulate the sub-G_1_ and S phase of HL-60 cells (Figure 4B). As a control, BBR did not show a significant effect on the cell cycle. These results suggested that Ber8 induced a cell cycle arrest in S and G_2_/M phase along with the inducement of a sub-G_1_ peak.

The accumulation of cells in the S-G_2_/M phase is usually due to the induction of a DNA damage and repair pathway [30]. For this reason, we examined the related pathway induced by Ber8 by using Western blot. As shown in Figure 4C and 4D, Siha and HL60 cells treated with Ber8 for 48 h induced a dose-dependent increase of phosphorylated ATM (p-ATM), phosphorylated p53 (p-p53) and phosphorylated H2AX (γH2AX). These findings indicated the happening of DNA damage and repair with the up-regulation of p-ATM and p-p53. Moreover, Ber8 decreased the primary transcription factor C-MYC in a dose-dependent manner. C-MYC can influence on the process of multistage cancer development, and its down-regulation can promote apoptosis and senescence [31]. Additionally, P21 and P27, the key downstream regulators of cell cycle arrest and cellular senescence [32, 33], were also increased by Ber8.

In addition, long-term treatments of Siha and HL-60 cells with Ber8 led to apparent senescence, with larger cell size, vacuolated cytoplasm, and β-galactosidase activity (Figure 4E). The percentage of SA-β-gal-positive cells reached the significant values of 71.9% and 91.4% in Siha cells and HL-60 cells, respectively (Figure 4F). However, BJ fibroblasts displayed a healthy, normal morphology after treatment with Ber8 and did not present any β-galactosidase activity. Together, these results demonstrated that the inhibition of cell proliferation and arrest of cell cycle by Ber8 were accompanied with the induction of a DNA damage, repair pathway, and cell senescence.

### The effects of Ber8 on telomeric G-quadruplex *in vivo*

**Figure 5:**
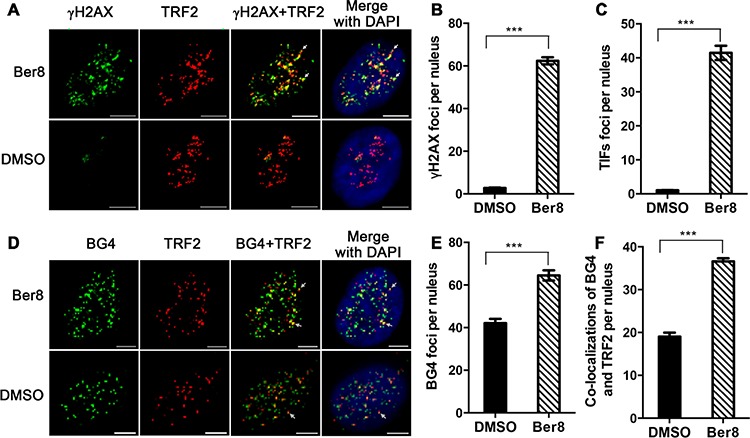
Ber8 stimulated DNA damage and stabilized endogenous G-quadruplexes at telomeric regions **A.** Representative immunofluorescence images of γH2AX (green) and TRF2 (red) foci in Siha cells treated with 2.5 μM Ber8 or 0.1% DMSO for 24 h (Original magnification, 40×; *bars*, 5 μm). The nuclei were stained with DAPI (blue), and typical co-localization foci were indicated by white arrows. **B.** Quantification of γH2AX foci number per nucleus. **C.** Quantification of TIF numbers per nucleus. **D.** Representative immunofluorescence images of BG4 (green) and TRF2 (red) foci in Siha cells treated with 2.5 μM Ber8 or 0.1% DMSO for 24 h (Original magnification, 40×; *bars*, 5 μm). The nuclei were stained with DAPI (blue), and typical co-localization foci are indicated by white arrows. **E.** Quantification of BG4 foci numbers per nucleus. **F.** Quantification of co-localization of BG4 and TRF2 per nucleus. In all experiments, >50 nuclei were counted in each group, and the s.e.m. was calculated from three replicates. ***, *P* < 0.0001 compared with DMSO.

G-quadruplex ligand PDS could induce DNA damage in telomeric region and non-telomeric DNA loci [34]. The previous results showed that Ber8 could bind with and stabilize telomeric G-quadruplexes, and induced DNA damage. We further performed double immunofluorescence experiments to investigate whether the DNA damage induced by Ber8 was at telomere region. A significant increase in γH2AX foci (with a mean of 62 foci per nucleus) was observed after treatment with Ber8 for 24 h (Figure 5A and 5B), indicating that Ber8 induced DNA double-strand breaks. To verify whether γH2AX was activated at the telomeres, we quantitatively analyzed the co-localization of γH2AX with TRF2, which formed the so-called telomere dysfunction-induced foci (TIFs) [35]. We found that treatment with Ber8 significantly increased the TIFs, with a mean of 41 TIFs per nucleus (Figure 5A and 5C). More importantly, about 66% of γH2AX foci were co-localized to TRF2. The TRF2 foci maintained in 24-h treatment (Supplementary Figure S3). These results suggested that the DNA damage stimulated by Ber8 occurred largely at the telomeric region.

Whether Ber8 could stabilize or change the number of endogenous telomeric G-quadruplexes was further investigated using the BG4 antibody, which was used for quantitative visualization of DNA G-quadruplexes in human cells [1]. Strikingly, 24-h treatment with Ber8 induced a significant increase of BG4 foci in the nucleus, with a mean of 65 BG4 foci per nucleus (Figure 5D and 5E), indicating that Ber8 could increase the amount of G-quadruplexes *in vivo*. Moreover, to track whether the compound stabilized telomeric G-quadruplexes, double immunofluorescence experiments were also performed to stain both BG4 and TRF2. As shown in Figure 5D and 5F, the co-localization of BG4 and TRF2 protein was considerably increased by Ber8 (with a mean of 37 foci per nucleus) compared with that of the control cells (with a mean of 19 foci per nucleus). Approximately 58% of BG4 foci were co-localized to TRF2, suggesting the inducement of endogenous telomeric G-quadruplex structures in living cells by Ber8.

### Ber8-induced dissociation of telomere-binding proteins and telomere uncapping

**Figure 6:**
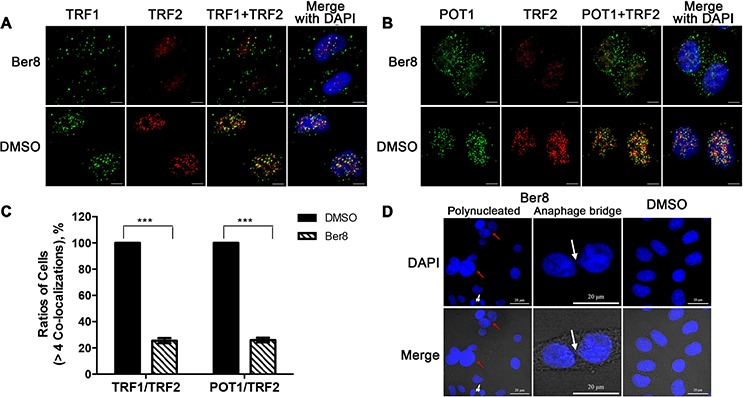
Dissociation of telomere-binding proteins and telomere uncapping induced by Ber8 **A.** Representative immunofluorescence images of TRF1 (green) and TRF2 (red) foci in Siha cells treated with 2.5 μM Ber8 or 0.1% DMSO for 48 h (original magnification, 40×; *bars*, 5 μm). The nuclei were stained with DAPI (blue). **B.** Representative immunofluorescence images of POT1 (green) and TRF2 (red) foci in Siha cells treated with 2.5 μM Ber8 or 0.1% DMSO for 48 h (original magnification, 40×; *bars*, 5 μm). The nuclei were stained with DAPI (blue). **C.** Percentage of cells with more than four co-localizations per nucleus of TRF1/TRF2 or TRF1/POT1. In total, >50 nuclei were counted in each group, and the s.e.m. was calculated from three replicates. ***, *P* < 0.0001 compared with DMSO. **D.** Representative images of polynucleated cells and anaphase bridges in Siha cells treated with 0.62 μM Ber8 for 16 days. The cells were stained with DAPI, and images were recorded (original magnification, 40×; *bars*, 20 μm). The red arrow indicated a typical image of polynucleated cells, and the white arrow indicated a typical image of anaphase bridge formation.

Considering that stabilization of the G-quadruplex in the telomeric region may result in the dissociation of telomere binding protein and telomere uncapping [36], we next investigated the effects of Ber8 on the dissociation of telomere-binding proteins, including POT1, TRF1 and TRF2, which directly recognized telomere TTAGGG repeats. Double immunofluorescence experiments demonstrated that TRF1 and POT1 expression highly overlapped with that of TRF2 in Siha control cells (Figure 6A and 6B). 48-h treatment with Ber8 specifically delocalized TRF1 and POT1 (but not TRF2) from the nucleus to the cytoplasm (Figure 6A and 6B). Quantitative analysis the percentage of nuclei with more than 4 TRF1/TRF2 and POT1/TRF1 co-localizations revealed a significant reduction to 25.4% ± 3.4% and 25.9% ± 3.1%, respectively, after Ber8 treatment (Figure 6C). The dissociation of hTERT was not observed in Ber8-treated cells (data not shown). These results demonstrated that Ber8 strongly dissociated POT1 and TRF1 proteins from the nucleus to the cytoplasm.

One current model proposes that telomeres form ‘a cap’ at the end of chromosomes [7] and that alterations in telomere protein binding may lead to telomere uncapping, with the formation of anaphase bridges and polynucleated nuclei [37–39]. After staining nuclei with DAPI, typical images of multiple or polynucleated nuclei were observed in Siha cells treated with 0.62 μM Ber8 for 16 days (Figure 6D). Specifically, 24.0% of the nuclei examined (*n* = 200) was observed in Ber8-treated cells, compared with 1% for the controls. Moreover, typical images of anaphase bridges were found in Ber8-treated cells (Figure 6D) at a proportion of 29.5% compared with 0% for the controls. All the above data supported our hypothesis that Ber8 could stabilize endogenous telomeric G-quadruplexes and lead to telomere DNA damage and telomere end uncapping.

## DISCUSSION

G-quadruplex-stabilizing small molecules derived from polycyclic alkaloid structures are potent telomere-stabilizing agents *in vitro* and induce senescence or apoptosis in a variety of cancer cell lines [40–42]. Compounds that contain polycyclic alkaloids often exhibit improved solubility and can facilitate salt formation, which are important for oral absorption and bioavailability [43]. Using the favorable polycyclic skeleton of berberine to our advantage, our group has developed a series of 9-substituted berberine derivatives to stabilize G-quadruplexes [26–29]. Here we took some further screening and mechanic studies basing on our in-house berberine derivatives library. Ber8 was found with a significant selective anti-tumor activity on several cancer cells. Since mechanic studies indicated the effects of Ber8 on cancer cells mainly through its binding with G-quadruplex at telomere region, one of the reasons for cellular selectivity might come from the different basal level of DNA damage in telomere region of cancerous cells and normal cells [44], or the basal level of G-quadruplex varies in tumorous cells and normal cells [2]. However, this hypothesis still needs further exploration.

We also found Ber8-induced dissociation of telomere-binding proteins from the telomeres and translocation of these proteins from the nucleus to the cytoplasm. These binding proteins include single-stranded binding protein (POT1) and double-stranded binding protein (TRF1). We were curious about whether Ber8 could affect conformational switch of double-stranded TTAGGG repeats. The result of S1 nuclease digestion assay was shown in Supplementary Figure S4. Double-stranded DNA (dsHTG21) could not be digested by S1 nuclease while G-quadruplex DNA (HTG21) could be digested. After incubation with Ber8, the dsHTG21 exhibited significant digested bands in a dose-dependent manner. The results indicated that G-quadruplex structure might be induced by Ber8 in the double-stranded region. This finding was consistent with one current model that stabilization of telomere G-quadruplex may lead to alterations of telomere protein binding and telomere uncapping and, thus, cause DNA damage at the telomere [12, 36]. However, shortening of telomere length was not observed (Supplementary Figure S5), which was similar to other G-quadruplex ligands, such as telomestatin [36], NiP [37] and RHPS4 [38]. Possible reasons for this may include the survival advantage of cells with drug-undamaged telomeres during culture passages or that telomere uncapping induced two important DNA repair pathways that mend broken chromosomes: homology-directed repair (HDR) and nonhomologous endjoining (NHEJ) [7, 45].

In summary, we found an effective anti-tumor berberine derivative Ber8 that demonstrated an effect on several cellular events, including cell cycle arrest, DNA damage and repair, and cell senescence. Furthermore, *in vitro* and *in vivo* studies indicated that this compound could bind with the G-quadruplex structure in telomere ends and, thus, cause the dissociation of shelterin proteins, which might be the main mechanism in the anti-tumor action of the compound.

## MATERIALS AND METHODS

### Synthesis and characterization

All chemicals were obtained from commercial sources unless otherwise specified. All HPLC purified oligomers were purchased from Invitrogen (China), and the concentration was determined from its absorbance at 260 nm based on its molar extinction coefficients (ε_260_). The structure, synthesis route and characterization of the compounds were listed in the supplementary data (Supplementary Figure S1 and Supplementary Scheme S1).

### Fluorescence resonance energy transfer (FRET)

FRET assays were performed following previously described methods. 5′-FAM and 3′-TAMRA dual labeled F21T (5′-d(GGG[TTAGGG]_3_)-3′) at a final concentration of 0.2 μM were incubated with diverse concentrations of compounds in Tris-HCl buffer (10 mM, pH 7.4) containing 60 mM KCl at 37°C for 1 h. The fluorescence melting curves were determined within a Roche LightCycler2^®^ real-time PCR machine. Fluorescence readings with excitation at 470 nm and detection at 530 nm were taken at intervals of 1°C from 37 to 99°C, with a constant temperature maintained for 30 s prior to each reading to ensure a stable value. Final analysis of the data was conducted using Origin 8.0 (OriginLab Corp.).

### Surface plasmon resonance (SPR)

SPR measurements were performed on a ProteOn^®^ XPR36 Protein Interaction Array System (Bio-Rad Laboratories, Hercules, CA) using a Neutr Avidin-coated NLC sensor chip. In a typical experiment, biotinylated HTG22 (5′-d(AGGG[TTAGGG]_3_)−3′) and duplex DNA (5′-T_9_CGAATTCGT_5_CGAATTCG-3′) were folded in a filtered and degassed running buffer (50 mM Tris-HCl, pH 7.4, 100 mM KCl). The DNA samples were then captured (∼1,000 RU) in flow cells, and a blank cell was set as a control. Ligand solutions (at 0.625, 1.25, 2.5, 5, 10, and 20 μM) were prepared within the running buffer by serial dilutions from stock solutions. Six concentrations were injected simultaneously at a flow rate of 25 μL/min for 5 min for associating, followed by 5 min of disassociation at 25°C. The NLC sensor chip was regenerated with a short injection of 1 M KCl between consecutive measurements. The final graphs were obtained by subtracting blank sensorgrams from the duplex or quadruplex sensorgrams. The data were analyzed with ProteOn^®^ manager software, using the Langmuir model to fit the kinetic data.

### Circular dichroism (CD) spectroscopy

A final concentration of 5 μM HTG21 (5′-d(GGG[TTAGGG]_3_)−3′) oligomers was re-suspended in a CD buffer (10 mM Tris-HCl, pH 7.4) with varying amounts of compounds and incubated for 5 min. The CD spectra were recorded on a Chirascan^®^ CD spectrophotometer (Applied Photo-physics, UK) at 25°C. A quartz cuvette with a 4-mm path length was used for the spectra recorded over a wavelength range of 230–400 nm at a 1-nm bandwidth, 1-nm step size, and 0.5-s per point. A buffer baseline was collected in the same cuvette and subtracted from the sample spectra. Final analysis of the data was conducted using Origin 8.0 (OriginLab Corp.).

### Cell culture

The human cervical cancer cell Siha, human lung cancer cell A549, human promyelocytic leukemia cell HL-60, and BJ fibroblasts were obtained from the American Type Culture Collection (ATCC, Rockville, MD) and preserved at our lab. HL-60 cells were cultured in a RPMI-1640 medium (Gibco, Carlsbad, CA) supplemented with 10% fetal bovine serum (Gibco, Carlsbad, CA), and other cell lines were grown in Dulbecco's modified Eagle's medium (D-MEM, Gibco Carlsbad, CA) supplemented with 10% fetal bovine serum. All cells were cultured with 5% CO_2_ at 37°C.

### Short-term cell viability

Cells were seeded on 96-well plates (5.0 × 10^3^cells / well) and exposed to various concentrations of compounds. After 48-h or 96-h treatment, 20 μL of 2.5 mg/mL methylthiazolyl tetrazolium (MTT) solution was added to each well, and the cells were further incubated for 4 h. The cells in each well were then treated with dimethyl sulfoxide (DMSO) (100 μL per well), and the optical density (OD) was recorded at 570 nm. All experiments were parallel performed in triplicate, and the IC_50_ values were derived from the mean OD values of the triplicate tests versus the drug concentration curves.

### Long-term cell culture

Long-term proliferation experiments were conducted using Siha, HL60, and BJ cells. The cells were grown in culture dishes at 5.0 × 10^5^ per dish and were exposed to compounds at subcytotoxic concentrations or an equivalent volume of 0.1% DMSO every four days. The cells in the control and drug-exposed dishes were counted, and the flasks were reseeded with 5.0 × 10^5^ cells. The process was repeated until a growth platform appeared.

### Senescence analysis

After completion of the long-term cell culture, the growth medium was aspirated, and the cells were fixed by 2% formaldehyde/0.2% glutaraldehyde for 15 min at room temperature. The fixing solution was then removed, and the cells were gently washed with PBS twice. The cells were then stained using β-Gal stain solution containing 1 mg/mL of 5-bromo-4-chloro-3-indolyl-β-D-galactoside and were incubated at 37°C overnight. The staining solution was then removed, and the cells were washed three times with PBS. The cells were viewed and photographed under an optical microscope.

### Flow cytometric analysis

The cells treated with compounds or control medium were washed in PBS and fixed with 70% ethanol, then centrifuged and re-suspended in a staining solution (50 μg/mL PI, 75 KU/mL RNase A in PBS) for 30 min at room temperature in dark. Cells were analyzed by flow cytometry using an EPICS XL flow cytometer (Beckman Coulter, USA). For each analysis, 13,000 events were collected. The cell cycle distribution was analyzed by EXPO32 ADC software.

### Western blotting

The cells treated with compounds or control medium were collected and lysed in RIPA lysis buffer (Bioteke, China), and the protein concentrations were determined using a BCA protein assay kit (Pierce, U.S.A.). In total, 60 μg of protein was resolved on a 12% SDS-PAGE and was transferred to 0.22-μm immobilon polyvinyl difluoride (PVDF) membranes. The blots were blocked with 3% BSA for 2 h at 25°C and then probed with primary antibodies (1:1,000) at 4°C for 16 h. After three washes, the blots were subsequently incubated with the corresponding secondary antibodies (1:3,000) for 2 h at 25°C. The protein bands were visualized using chemiluminescence substrate, and images were acquired using a Tanon-4200SF gel imaging system (Shanghai, China). Antibodies to β-actin (#4970, Cell Signaling Technology, MA, USA), Phospho-ATM (Ser1981) (#5883, Cell Signaling Technology), Phospho-p53 (Ser15) (#9286, Cell Signaling Technology), γH2AX (#9718, Cell Signaling Technology), C-MYC (#9402, Cell Signaling Technology), P21(#2947, Cell Signaling Technology), P27(#3686, Cell Signaling Technology), anti-rabbit IgG-HRP (#7074, Cell Signaling Technology), and anti-mouse IgG-HRP (#7076, Cell Signaling Technology) were used.

### Immunofluorescence

Cells grown on glass coverslips were fixed in 4% paraformaldehyde/PBS for 15 min, then permeabilized with 0.1% triton-X100/PBS at 37°C for 30 min, and finally blocked with 5% goat serum/PBS at 37°C for 3 h. Immunofluorescence was performed using standard methods, and the slides were incubated alternately with BG4 (80 ng/μL), anti-FLAG antibody (#2368, Cell Signaling Technology), γH2AX antibody (#9718, Cell Signaling Technology), TRF2 antibody (ab13579, Abcam), or POT1 antibody (ab21382, Abcam) at 37°C for 3 h. The glass coverslips were washed six times with blocking buffer and were then incubated with anti-rabbit Alexa 488-conjugated antibody (A21206, Life Technology), anti-mouse Alexa 555-conjugated antibody (A21427, Life Technology), and 2 μg/mL of 4′,6-diamidino-2-phenylindole (DAPI, Invitrogen) at 37°C for 3 h. The glass coverslips were again washed six times with blocking buffer, and then, digital images were recorded using an LSM710 microscope (Zeiss, GER) and analyzed with ZEN software. Fifty nuclei were counted in each group, and the s.e.m. was calculated from three replicates. Frequency distribution graphs were plotted using GraphPad Prism (GraphPadSoftware Inc.).

### Anaphase bridge analysis

After the long-term cell culture, the growth medium was aspirated, and the cells were fixed in 2% formaldehyde/0.2% glutaraldehyde for 15 min at room temperature. The fixing solution was removed, and the cells were gently washed twice with PBS and then stained with 2 μg/mL of DAPI at 37°C for 3 h. The cells were washed with PBS twice, and digital images were recorded using LSM710 microscope (Zeiss, GER) and analyzed with ZEN software. The frequency of anaphase bridges was calculated as the ratio between cells exhibiting anaphase bridges and the total number of cells. A minimum of 50 anaphase cells were examined in each experiment.

## SUPPLEMENTARY MATERIAL FIGURES AND TABLES



## Abbreviations

BBR: berberine
hTERT: telomerase reverse transcriptase
POT1: protection of telomeres 1
TRF1 and TRF2: telomeric repeat binding factors 1 and 2
MTT: methylthiazolyltetrazolium
DAPI: 4′,6-diamidino-2-phenylindole
DMSO: dimethylsulfoxide
FRET: fluorescence resonance energy transfer
SPR: surface plasmon resonance
CD: circular dichroism.
