# Supplementary material for: A newly identified berberine derivative induces cancer cell senescence by stabilizing endogenous G-quadruplexes and sparking a DNA damage response at the telomere region
Source: Oncotarget. 2015 Oct 8;6(34):35625–35. doi: 10.18632/oncotarget.5521 (PMC4742130; doi:10.18632/oncotarget.5521)
Supplement: Supplementary file 1 [file oncotarget-06-35625-s001.pdf]

# SUPPLEMENTARY METHODS

## General synthetic chemistry

The compounds ber1-ber22 were prepared in our previous synthesis except ber8[1–4]. Reactions were carried out under nitrogen when necessary. All the material we used were purchased from commercial suppliers and used without further purification. Solvents were dried over Molecular sieves type 4A. Flash silica chromatography was performed using silica gel (200–300 mesh), dichloromethane/methanol (20/1). Reactions were routinely monitored by thin-layer chromatography on silica gel $GF_{254}$ and visualized with ultraviolet lamp (254 nm or 365 nm). $^{1}H$ and $^{13}C$ NMR spectra were determined on a BrukerAvance III 400 spectrometer using TMS as an internal standard in DMSO-$d_6$ solution, operating at a frequency of 400 MHz for proton and 101 MHz for carbon. Peak positions are given in parts per million ($\delta$) from tetramethylsilane, and coupling constant value ($J$) are given in Hz. High-resolution mass spectra were measured on an Shimhdzu LCMS-IT-TOF instrument with an ESI mass selective detector in positive ion mode. The purities of synthesized compounds were confirmed to be higher than 95% by using analytical HPLC equipped with an Analaric C18 column (150 × 4.6 mm, 5 µm particle size) and eluted with methanol/water (70:30) containing 0.1% TFA at a flow rate of 0.4 mL/min, with calculation of the relative purity of each compound based on its absorption at 254 nm.

The scheme of synthesis of the Berberine derivative Ber8 (3) was shown in Supplementary Scheme S1.

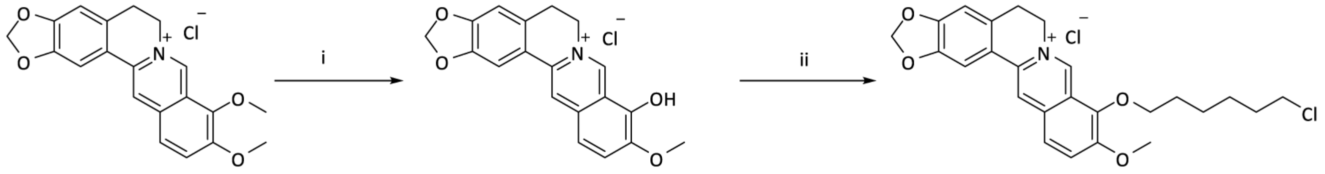


**Supplementary Schemes S1: Reagents and conditions: (i) 190°C, 3 h; (ii) $Cl(CH_2)_6Cl$, $CH_3CN$, 140°C, 20 h.**

The detailed procedure for the synthesis of Ber8 was shown as follows.

## Preparation of berberrabine (2)

The protocol used by Yan Ma et.al. [3] was adapted with minor modifications. A solution of berberine **1** (5 g, 13 mmol) in decalin was stirred at 190°C under nitrogen for about 3h. The resulting brownish red solid was collected by filtration, washed with n-hexane and dried under infrared lamp for 2 h. Berberrabine (**2**) was obtained as brownish red solid (4.1g, 85%). $^{1}H$ NMR (400 MHz, DMSO-*d6*) $\delta$ 9.08 (s, 1H), 7.98 (s, 1H), 7.61 (s, 1H), 7.21 (d, $J$ = 7.9 Hz, 1H), 6.96 (s, 1H), 6.35 (d, $J$ = 7.8 Hz, 1H), 6.10 (s, 2H), 4.48 (t, $J$ = 6.0 Hz, 2H), 3.72 (s, 3H), 3.07 – 2.99 (m, 2H).

## Preparation of 9-O-6-ammonia chloride hexylberberine (Ber8)

The protocol used by David A. Walsh et.al. [5] was adapted with minor modifications. To a 75 mL sealed tube was added berberrabine**2** (500 mg, 1.4 mmol), 1,6-dichlorohexane (1.6 mL, 11 mmol, 8 equiv) and dry acetonitrile (50 mL). The reaction mixture was stirred at 140°C for 20 h, cooled and concentrated *in vacuo*. The residue was purified by flash chromatography ($SiO_2$, dichloromethane/methanol, 20/1) to afford **3** as wheat solid (225mg, 35%). $^{1}H$ NMR (400 MHz, DMSO-$d_6$) $\delta$ 9.77 (s, 1H), 8.97 (s, 1H), 8.20 (d, $J$ = 9.2 Hz, 1H), 8.00 (d, $J$ = 9.1 Hz, 1H), 7.81 (s, 1H), 7.10 (s, 1H), 6.18 (s, 2H), 4.97 (t, $J$ = 6.1 Hz, 2H), 4.29 (t, $J$ = 6.7 Hz, 2H), 4.06 (s, 3H), 3.67 (t, $J$ = 6.6 Hz, 2H), 3.25 – 3.18 (m, 2H), 1.94 – 1.84 (m, 2H), 1.82 – 1.72 (m, 2H), 1.56 – 1.45 (m, 4H). $^{13}C$ NMR (100 MHz, DMSO-$d_6$) $\delta$ 150.9, 150.3, 148.2, 145.8, 143.3, 137.9, 133.5, 131.2, 127.1 (s), 123.8, 122.1, 121.0, 120.7, 108.9, 105.9, 102.6, 74.6, 57.5, 55.8, 45.9, 32.5, 29.8, 26.8, 26.6, 25.0. HR LC-MS: *m/z*calcd for $C_{25}H_{26}NOCl$: 440.1623$[M+H]^+$; found: 440.1633.

## Cellular uptake

Cells were seeded at a density of 5 × $10^6$ cells / 10 cm dish in 10 mL of culture medium on day 1. On day 2, 5 µM of **Ber8** or 0.1% DMSO was added into the cells. After 48 h of drug exposure, cells were collected and washed with PBS twice. 1 × $10^6$ cells were lysis by 500 µL RIPA lysis buffer (Bioteke, China) and the supernatant was collected. The UV absorbance of the compound was

detected at 25°C by using UV-2450 spectrophotometer (Shimadzu, Japan). The optimal wavelengths were 200–500 nm. To determine the drug concentration, calibration curves in the concentration range of 0.05–32 μM in RIPA lysis buffer were prepared.

### S1 nuclease digestion assay

The 5'-FAM-labeled HTG21 (5'-FAM-GGGTTAGGGTTAGGTTAGGG-3') and non-labeled HTC21 (5'-CCCTAACCCTAACCCT-AACCC-3') were dissolved in 10 mM Tris-HCl (pH 7.4, 100 mM KCl) with a final concentration of 100 μM. 100 pmol of the HTG21 oligomer was annealed by heating to 95°C for 10 min then gradually cooled to room temperature for G-quadruplex formation, and 100 pmol of the HTG21 and HTC21 oligomers were annealed together using the same procedure for duplex formation. Samples were incubated with Ber8 at various concentrations (100, 500, and 2500 pmol) at 37°C for 1 hour. 1.5 U of S1 nuclease was then added to each sample and incubated at 37°C for 5 min. The digestion was terminated by adding 50 mM EDTA then incubated at 70°C for 10 min letting the nuclease denature. Sample was resolved in 16% native gel in 0.5 × TBE.

### Telomere length assay

Long-term cultured cells were treated with Ber8. Telomere length was measured using Telo TAGGG Telomere Length Assay KIT (#2209136, Roche). Briefly, genomic DNA was digested with Hinf I/Rsa I restriction enzymes. The digested DNA fragments were separated on a 0.8% agarose gel, transferred to a nylon membrane, and fixed DNA on a wet blotting membrane by baking the membrane at 120°C for 20 min. Membrane was hybridized with a DIG-labeled hybridization probe for telomeric repeats and incubated with anti-DIG-alkaline phosphatase. TRF was performed by chemiluminescence detection.

## REFERENCES


1. Zhang WJ, Ou TM, Lu YJ, Huang YY, Wu WB, Huang ZS, Zhou JL, Wong KY, Gu LQ. 9-Substituted berberine derivatives as G-quadruplex stabilizing ligands in telomeric DNA. Bioorganic & medicinal chemistry. 2007; 15:5493–5501.
2. Ma Y, Ou TM, Hou JQ, Lu YJ, Tan JH, Gu LQ, Huang ZS. 9-N-Substituted berberine derivatives: stabilization of G-quadruplex DNA and down-regulation of oncogene c-myc. Bioorganic & medicinal chemistry. 2008; 16:7582–7591.
3. Ma Y, Ou TM, Tan JH, Hou JQ, Huang SL, Gu LQ, Huang ZS. Synthesis and evaluation of 9-O-substituted berberine derivatives containing aza-aromatic terminal group as highly selective telomeric G-quadruplex stabilizing ligands. Bioorganic & medicinal chemistry letters. 2009; 19:3414–3417.
4. Ma Y, Huang ZS. Synthesis and activity evaluation of 9-O-substituted berberine derivatives containing polyamine chain as highly selective telomeric G-quadruplex stabilizing ligands. Chem J of Chinese Universities. 2012; 33:2217–2222.
5. Walsh DA, Franzyshen SK, Yanni JM. Synthesis and antiallergy activity of 4-(diarylhydroxymethyl)-1-[3-(aryloxy) propyl]piperidines and structurally related compounds. Journal of medicinal chemistry. 1989; 32:105–118.

## SUPPLEMENTARY FIGURES AND TABLES

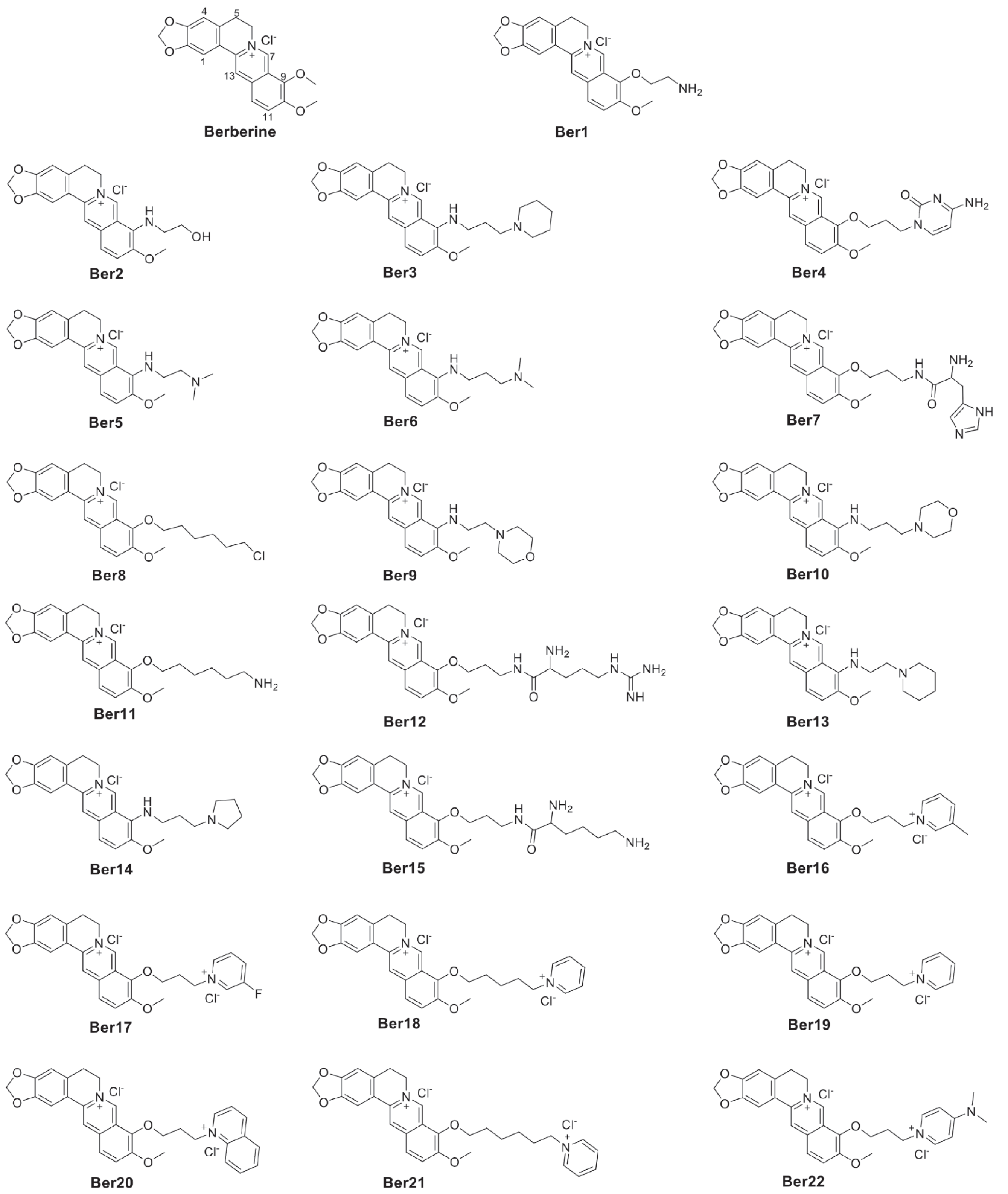


**Supplementary Figure S1: Structures of berberine and berberine derivatives Ber1~Ber22.**

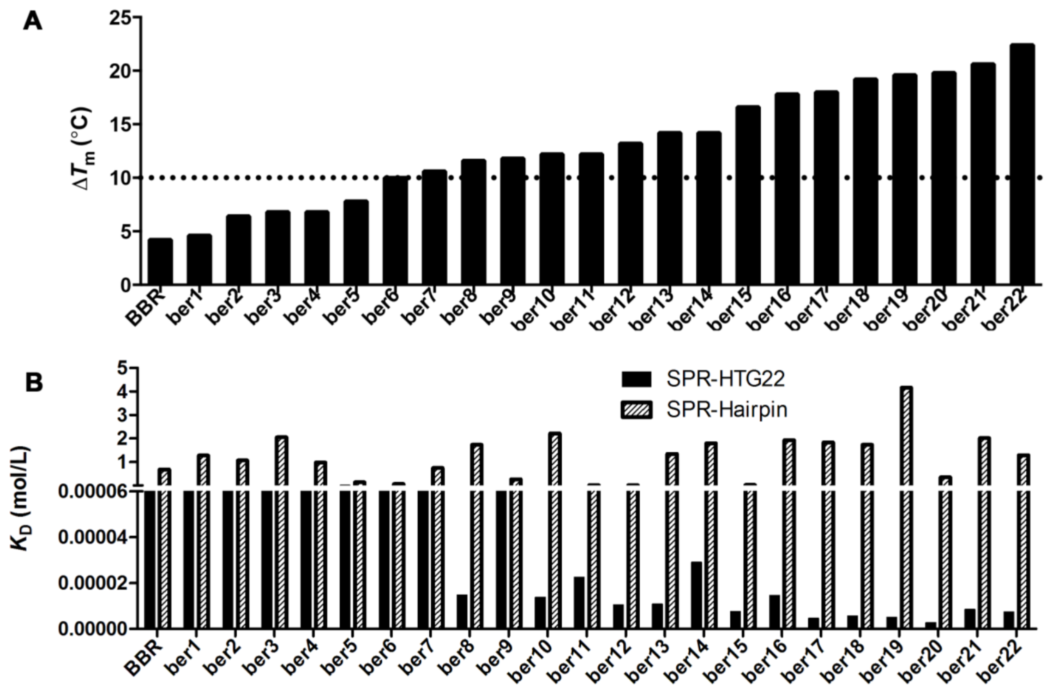


**Supplementary Figure S2: Stabilization and binding ability of berberine derivatives with telomere G-quadruplexes. A.** The changes in melting temperatures ($\Delta T_m$) of G-quadruplexes in the addition of different compounds by using FRET assay. Concentrations of F21T were 0.2 μM and compounds were 1 μM. In the absence of compounds, the $T_m$ value of annealed F21T was 60°C. $\Delta T_m = T_m$ (DNA + ligand) – $T_m$ (DNA). **B.** The equilibrium dissociation constant ($K_D$) of compounds bound to the HTG22 or the hairpin DNA by using SPR assays. The y-axis was set as a two-segment axis to better view the data.

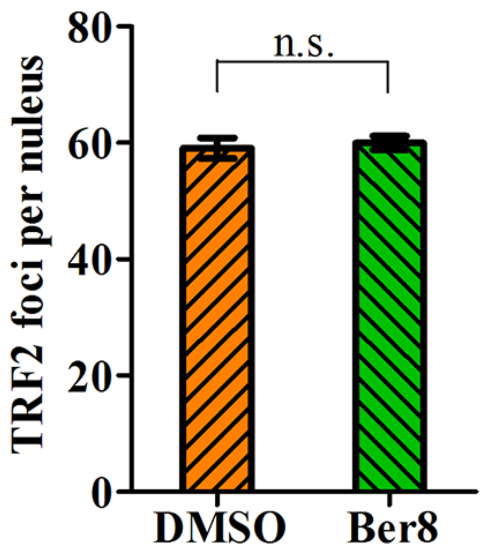


**Supplementary Figure S3: Quantification of TRF2 foci number per nucleus.** Siha cells were treated with 2.5 μM Ber8 or 0.1% DMSO for 24 h. Over 50 nuclei were counted in each group and the s.e.m. was calculated from three replicates.

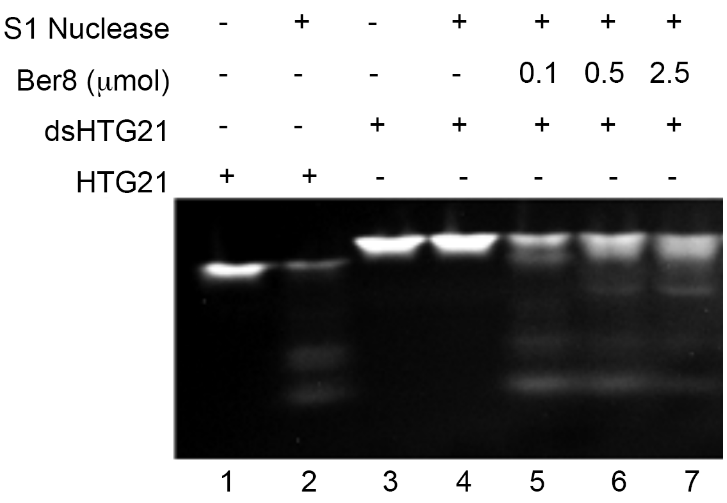


**Supplementary Figure S4: Detection of telomeric duplex conformation change by using S1 digestion assay.** 100 pmol of DNA was used for each sample, and was incubated with Ber8 on various concentrations (0.1, 0.5, and 2.5 μmol) at 37°C for 1 hour, then each sample was digested by 1.5 U of S1 nuclease at 37°C for 5 min and resolved on a 16% native PAGE gel. Digestion of HTG21 G-quadruplex was set as a positive control (line 2) and digestion of double-strand HTG21 was set as a negative control (line 4).

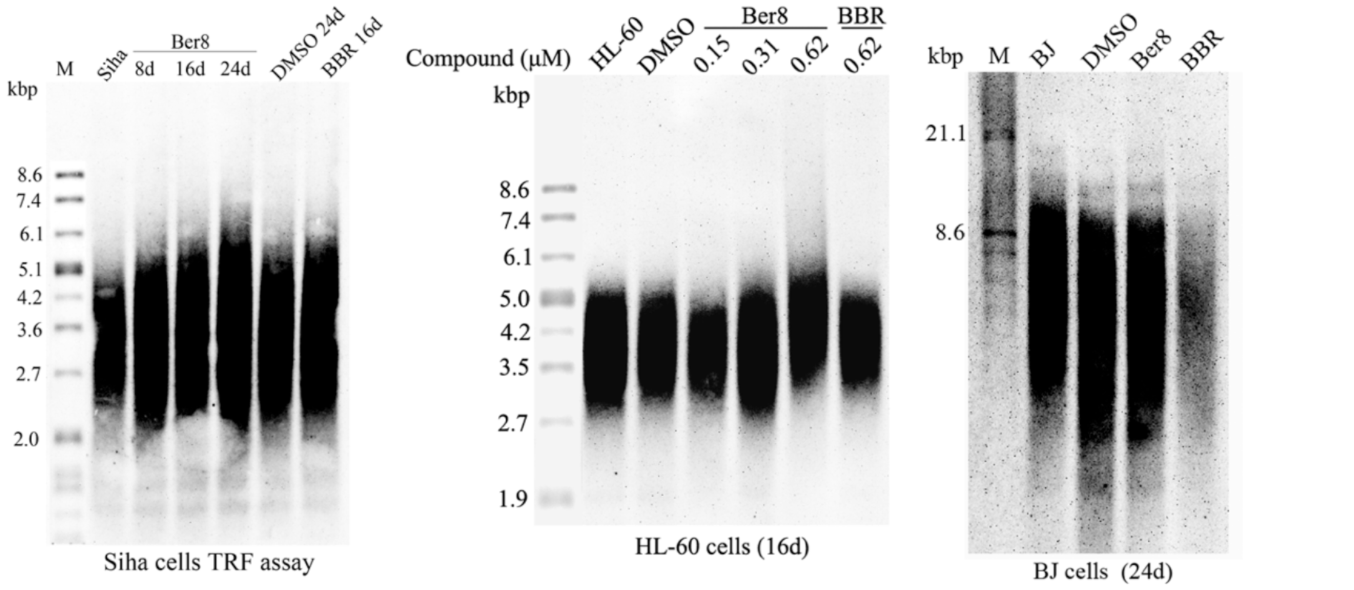


**Supplementary Figure S5: Effect of Ber8 on telomere length.** TRF of cancer cells treated or untreated with Ber8 was analyzed using the Telo TAGGG telomere length assay.

**Supplementary Table S1: The binding constants ($K_D$, mol/L) of compounds to the HTG22 oligomer or the hairpin DNA in SPR assays**

| | with HTG22 | with hairpin | | with HTG22 | with hairpin |
|---|---|---|---|---|---|
| **BBR** | 1.7E-04 | 0.67 | **ber12** | 1.05E-05 | 1.39E-02 |
| **Ber1** | 1.33E-04 | 1.27 | **ber13** | 1.08E-05 | 1.35 |
| **Ber2** | 6.80E-04 | 1.07 | **ber14** | 2.90E-05 | 1.8 |
| **Ber3** | 1.49E-04 | 2.05 | **ber15** | 7.57E-06 | 2.89E-02 |
| **Ber4** | 5.96E-04 | 9.80E-01 | **ber16** | 1.46E-05 | 1.93 |
| **Ber5** | 6.89E-03 | 1.37E-01 | **ber17** | 4.58E-06 | 1.83 |
| **Ber6** | 4.46E-04 | 6.35E-02 | **ber18** | 5.61E-06 | 1.73 |
| **ber7** | 5.33E-04 | 0.75 | **ber19** | 5.10E-06 | 4.16 |
| **Ber8** | 1.48E-05 | 1.73 | **ber20** | 2.75E-06 | 0.35 |
| **ber9** | 1.23E-04 | 0.26 | **ber21** | 8.53E-06 | 2.01 |
| **ber10** | 1.38E-05 | 2.21 | **ber22** | 7.37E-06 | 1.29 |
| **ber11** | 2.26E-05 | 1.75E-02 | | | |

**Supplementary Table S2: Inhibitory effects and cellular uptake of Ber8 in different cell lines***

| Cell line | $IC_{50}$ (μM) | cellular uptake (pmol/$10^6$ cells) |
|---|---|---|
| HL-60 | 1.7 ± 0.5 | 857.0 ± 10.3 |
| Siha | 6.0 ± 0.9 | 700.0 ± 39.2 |
| A549 | 18.6 ± 1.3 | 619.7 ± 25.4 |
| BJ | 28.8 ± 1.0 | 661.9 ± 17.1 |

* $IC_{50}$ values (the Ber8 concentration in inhibiting cell growth by 50% after 48 h incubation) were read from inhibitory curves that were fitted by exponential equation. Cellular uptake was performed with 5 μM Ber8. Each value represented the mean ± SD in three independent experiments.

**Supplementary Table S3: Time-dependent growth inhibition by BBR and Ber8 in MTT assay**

| $IC_{50}$ (μM) | | Siha cells | HL-60 cells | A549 cells | BJ cells |
|---|---|---|---|---|---|
| **Ber8** | 48 h | 6.0 ± 0.9 | 1.7 ± 0.5 | 18.6 ± 1.3 | 28.8 ± 1.0 |
| | 96 h | 5.4 ± 0.6 | 1.6 ± 0.6 | 2.5 ± 0.8 | 18.0 ± 0.6 |
| **BBR** | 48 h | 25.7 ± 1.1 | 6.5 ± 0.5 | >40 | 39.5 ± 0.8 |
| | 96 h | 33.6 ± 0.9 | 5.7 ± 0.6 | 18.0 ± 0.9 | 25.3 ± 0.7 |
